# Fever in Great Britain, &c.

**Published:** 1819-07-01

**Authors:** 


### FEVER IN GREAT BRITAIN, &c.

### VII

*Reports of the Practice in the Clinical Wards of the Royal Infirmary of Edinburgh, during the Months of November, December, and January,* 1817—18; *and May, June, and July,* 1818. By ANDREW DUNCAN, M. D. &c. &c. 8vo. pp. 143. Edinburgh and London, 1818.

### VIII.

*Observations on the Prevention and Treatment of the Epidemic Fever, at present prevailing in this Metropolis and most Parts of the United Kingdom, &c. &c.* By HENRY CLUTTERBUCK, M. D. &c. 1 Vol. 8vo. pp. 299. London, 1819.

### IX.

*Practical Observations on the Treatment and Prevention of Typhous Fever.* By EDWARD PERCIVAL, M. B. &c. 1 Vol. 8vo. pp. 156. London, 1819.

### X.

*Uber den anstechenden Typhus.* Von J. V. EDLER, Von HILDEBRAND, &c.

IN our last number we gave some account of the latest publications on the subject of the prevailing epidemic fever, as it appears in the sister kingdom; it is our duty now to present a brief sketch of the most recent works on the epidemic fever of England and Scotland. To this we shall proceed, without preface or preliminary remarks; our analyses being guided by the order of time, at which the different publications appeared.

Dr. Duncan, who is well known in the medical world, not only as a professor of medical jurisprudence in the University of Edinburgh, but as Editor of the first medical journal in Europe, was induced to publish the following reports, in consequence of the late parliamentary enquiry into the state of fever, and a regret often expressed to him, that while from the hospitals of Dublin, Cork, Glasgow, and London, excellent descriptions of the present epidemic had been communicated to the public, no account of it had issued from the Royal Infirmary of Edinburgh.

Dr. Duncan states, that although a larger proportion of fever cases was admitted in winter than in summer, yet no regular declension or increase of the epidemic has been observed; the fever prevailing as much during a summer of almost tropical temperature, as during a raw and cold winter. "In both seasons its character, as referable to typhus or synochus, was nearly alike." It appeared to affect both sexes equally, in respect to number, but not so in mortality; very few females died. It spared the upper ranks much; but when once they came under fever, they suffered more severely than the lower orders of society. Our author concludes from this, that the higher classes either were less exposed to the exciting causes, or were more able to resist their influence; but, that when once they did operate, their action was more violent.

The contagious nature of the northern epidemic Dr. Duncan considers as being fully established by universal experience. And he asserts, that even where fevers did not spring from contagion themselves, they occasionally became capable of propagating the disease to others.

"I have not used, says Dr. D. Cullen's distinction of synochus and typhus, because I do not believe that the distinction exists in nature. I have never seen an instance of typhus fever according to his definition. All our severe fevers begin with excitement, and terminate in debility, or are instances of synochus; although in truth, they are the identical disease from which Cullen drew his description of typhus, and are genuine examples of the only typhus fever which exists." 27.

Our author has preferred distinguishing the cases by the epithets cephalic, pulmonic, gastric, enteritic, hepatic, &c. from the principal organs affected. "For although frequently the *functions of all* were somewhat disturbed, the force of the disease seemed generally to bear upon one or two, and sometimes upon different organs in succession." We should think that this alone was a sufficient reason for selecting some more appropriate appellation than that chosen; for at this rate, what name could be given to a fever where the brain, stomach and liver, might be affected? Cerebro-gastro-hepatic?

In almost every case the head was greatly affected in the beginning with intolerantia lucis et soni, succeeded by torpor, low delirium, or maniacal excitement. The rest of the nervous system was affected at the same time. "In the first period severe pain was felt along the whole course of the spine, especially at the nape of the neck, and at the sacrum; the limbs were sore, as if bruised, and often a rheumatic or paralytic state of them remained for some time after the fever had terminated." It is, indeed, wonderful that we have so long overlooked this train of symptoms along the spinal brain, so constant in all idiopathic fevers: we hope, however, that the attention of the medical world will be directed to it in future, with a profitable result.

The lungs were very commonly affected. At the beginning of the fever, the cough often greatly aggravated the head-ache, but sometimes the pulmonic symptoms did not supervene till the cephalic symptoms remitted.

"I am convinced, says Dr. Duncan, that the disease described by some authors as pneumonia typhodes, and stated to have been at times epidemic, was in fact continued fever, with great affection of the lungs."

In simple pneumonia, the fever is symptomatic of the local affection, and declines, *pari passu*, as this last is relieved; while in typhus, the local affection grows out of the general fever, the latter continuing its course after the former is cured. This reasoning applies to general fever with other local affections; and militates strongly against the doctrine of idiopathic fever being dependant on the local inflammation and congestion.

In every case the stomach was more or less affected from the first, as indicated by anorexia, nausea, vomiting, or pain at the epigastrium, which was generally tender or painful to the touch. At the same time the functions of the intestines were seldom deranged, so that few cases came under the description of enteritic fever. The catamenia were not interrupted by the fever; on the contrary, they returned in several cases where they had previously been suppressed for some months. The liver was less frequently affected than, from the description of some epidemics, might have been expected. In many cases the principal complaint of the patient was of pains in various parts of the body, and loss of power, especially of the lower limbs. In several cases there were petechiæ, or an exanthematous efflorescence on the skin, neither of them, however, connected with any remarkable severity of the disease.

In most instances the strength and frequency of the pulse was increased, during the existence of the fever; in some, frequent and weak; in others, full, but natural as to frequency; in some it was almost natural, or only weak; and in many these circumstances varied during the course of the disease; and, contrary to the common idea of synochus, the pulse sometimes became fuller and stronger in the progress of the disease.

In one case, an excessive action of the heart and arteries was suspended by the fever, but returned after the fever was over. In the same manner, a very obstinate tympanites disappeared, during the fever, and afterwards recurred. The skin was parched in almost every case, till after the crisis. The temperature was, in most instances, increased; in summer often reaching as high as 104 or 5 in the axilla. In one fatal case, the temperature continued at 103 for some hours after death. The general ratio of mortality was 1 in 12 or 13, but the scale in the infirmary was too confined to warrant any general conclusions on this head. Some interesting dissections are given in the Appendix, in which the head appeared to be the organ which bore the greatest onus of the disease.

The Treatment of the patients under Dr. Duncan's care, was purely antiphlogistic, and he thinks it was probably owing to this circumstance that so little of that debility was observed, which is so common in these fevers when treated with stimulating and tonic remedies.

"So far as my observation goes, says this judicious physician, the debility which occurs in typhus fevers, is always the consequence of, and proportioned to, the previous excitement; and by reducing the violence of that excitement, in the early stages of the disease, by depletion, and the removal of every irritation, we prevent the debility from coming on, at least in the same degree." 33.

Some of the patients were able to get out of their beds almost as soon as the fever had run its course, and in none was there any sloughing of the nates from long pressure, which used frequently to carry off patients treated with stimulants, long after the fever had terminated. Opium was given only as an anodyne or diaphoretic, and very sparingly; wine but occasionally, and in small quantities, during the convalescence, and porter sometimes during the same period. The patients themselves acknowledged that they recruited faster with slops and ordinary diet than with generous food.

Blisters were the only *stimulants* (a term which we conceive to be a very improper one for counter-irritants) employed by Dr. D., and these often were of essential service, especially where the lungs and head were much affected.

The antiphlogistic treatment was generally very simple. In some cases, the saline mixture, with cold or tepid washing, laxatives, or enemas, when necessary, and an anodyne antimonial at night, answered perfectly well. In others a much more active plan was pursued; blood was freely abstracted, both locally and generally, and drastic purges liberally exhibited, with the most decided advantage.

"I cannot look back, says Dr. Duncan, upon the treatment of typhus, in the days of my apprenticeship, without wonder. In those days we, the students, would have shuddered if our teacher had prescribed blood-letting to a fever patient, as if he had ordered him to be put to death; and I have seen the exhibition of a simple saline purgative excite our severe censure. Nor did the amelioration of the patient after its operation gain any credit to our teacher. In our opinion every evacuation must be injurious in a disease of indirect debility." 35.

When there was great excitement of the whole arterial system, with intense head-ache, pain of neck and loins, suffused eyes, intolerantia lucis, pain at the pit of the stomach or belly, difficulty of breathing and a full throbbing pulse, venesection was preferred, and it was repeated at a short interval. At first our author contented himself with bleedings of eight ounces, but afterwards he became more bold, and frequently ordered twenty ounces to be taken at once. He never saw occasion to regret having taken too much, but often of having taken too little. He never ventured, however, to abstract forty ounces at a time, as was practiced by others. When the head was principally affected, arteriotomy was sometimes ordered; but where local bleeding was indicated, leeching or cupping was preferred. The relief obtained by abstraction of blood was often instantaneous and striking: the head-ache was removed as if by a charm; and, in some instances, permanently. Our author, however, was not so fortunate as to cut short the fever *in toto* by venesection alone. The violence of the disease was sometimes thus subdued, and it afterwards ran its course gently and quietly, the patient scarcely seeming more than languid and drowsy More commonly, however, the relief was only temporary, and in a day or two the head ache returned, though with less severity, and then it generally yielded to leeching the forehead.

"Although blood-letting was most beneficial in the early stages of our fever, there was no period of its course, nor indeed of the convalescence, in which it was not occasionally employed, when circumstances seemed to indicate its propriety." 36.

The cold affusion was very generally applied, and its good effects, in reducing morbid temperature, and alleviating the burning sensations of the patient were unequivocal; but Dr. Duncan has no confidence of its power in cutting short the fever, when the morbid catenation is once formed. In cases where the patient suffered from rigors or coldness of the extremities, the pediluvium was of the greatest benefit. Laxatives and evacuant enemas were also powerful auxiliaries; but our author seldom attempted to counteract the febrile excitement by drastic purging; and had " some fears that the function or structure of the intestines might be deranged by acting upon them too powerfully during a disease which is apt to direct its attack upon any weakened organ."

Emetics were not employed, but small doses of antimony or ipecacuan were combined with saline solutions. Mercury was sometimes ordered as a purgative, in the form of calomel, or the blue pill, but no trial was given to what is called the mercurial practice.

Dr. Duncan's work, like most modern medical publications, consists of one third text, and two thirds appendix; the latter, of course, being cases in detail. We cannot but fear that the present rage for composing works almost entirely of *cases*, may prove very prejudicial to medical literature, as it multiplies books with prodigious speed, and with scarce any labour. A volume of cases, if it be ever read through, will assuredly never be read a second time; whereas a well digested train of pathological, etiological, and therapeutical observations, interspersed with a few cases in illustration, would be placed in the library for constant reference, and continue as a class book for future generations. We appeal to those works which descend along the stream of time, edition after edition, for the truth of these remarks. They are not upheld by detailed cases, but by patient investigation, laborious research, or intense thought.*

It is but justice to say, that *Reports from Hospitals* are, in some measure, exempt from these strictures, since they form the most authentic and faithful documents that can possibly come before the medical public, and are therefore

---

* " A detail of cases does not appear to me, on the whole, the most instructive method of conveying to the public those results of experience on a large scale, which should rather exhibit the principles and conclusions of a well-furnished medical judgment." *Dr. Percival on Typhus Fever*, p. 119.

deserving of record in any shape. But we would suggest the propriety of adopting the plans pursued by our brethren in Ireland, as seen in the Dublin Hospital Reports, and Transactions of the Irish Associations. In respect to Dr. Duncan's little volume, it certainly contains a series of the most interesting cases which we have for a long time seen, in a similar space; not confined to fever alone, but including many other diseases, with dissections, and useful observations.

We now come to Dr. Clutterbuck's work, of which we shall be able to give but an imperfect account, because it principally consists of the old worn-out arguments that fever is inflammation of the brain. Dr. Clutterbuck seems determined to carry his point; for although every argument which he has used, has been refuted over and over again, while not a single writer, of any note, as far as we know, has adopted his theory, he repeats the very same story with some variations, and resolutely maintains his doctrines, in opposition to the facts, observations and dissections, brought forward by such numerous and respectable authorities. We shall, therefore, dip somewhat deeply into the subject of proximate causes, endeavouring, at the same time, to glean from the work as much *useful* information as we possibly can, leaving the ultimate decision, in a great measure, to *time*, which, indeed, seems far advanced in the demolition of our author's theory.

Dr. Clutterbuck justly observes, that fever is subject to so much variety in its *minute* phenomena, as to render very *minute* descriptions at least unnecessary. A family likeness however pervades the whole. Epidemic fevers particularly are different in different climates and seasons. Sydenham never saw two alike. Instead, therefore, of attempting to furnish a minute detail of all the varieties that have occurred in the present epidemic, Dr. C. contents himself with pointing out the leading and *essential* characters of the disease. Dr. Clutterbuck states, that after the most attentive observations, he has not been able to discover any difference in symptoms, whether the disease obviously originated in contagion, or arose independently of such a cause.

"I have observed the fever to follow exposure to some common cause of disease, such as cold, fatigue, or intemperance; and where not the least reason could be discovered for even suspecting it to proceed from *contagion*; yet the disease has afterwards proved infectious to others; and, in both cases, the symptoms have been quite undistinguishable."

Thus far we coincide with Dr. Clutterbuck, but demur to the following position.

"If this be really the case, it *predisposes* one to believe that the circumstance of contagion will have no material influence on the effects of remedies."

In the first place, we should not allow ourselves to be *predisposed* to beliefs, when *practical* decisions can be obtained. And our experience has long since taught us that a fever from cold or intemperance is infinitely more under the influence of medicines than one from contagion. We have rarely seen a fever of the latter kind *arrested* in its course, even by the most vigorous measures; whereas fevers from other causes will be frequently checked, even in the height of their career, by copious depletion. Our readers will observe a nearly similar remark a few pages back, by Dr. Duncan of Edinburgh.

We shall now proceed to examine Dr. Clutterbuck's symptomatology of fever, on which he mainly rests his doctrine of cerebral inflammation.

"The *first* symptom, says he, almost invariably *complained of*, is more or less of uneasiness in the head; sometimes trifling in degree, but often amounting to severe headache; when a pain is often felt, at the same time, in the eye balls, and which is aggravated whenever the eyes are turned to one side or the other." 15.

This passage adds another proof to the innumerable ones on record, how completely theorists are blinded to every object which militates against their darling hypotheses, however plain and conspicuous that object may be to every other and unprejudiced eye! Head-ache the *first* symptom! We assert that it is *not* the first symptom. Boerhaave, who studied the phenomena of fever with as much industry and talent as any man, selected *three* symptoms as the most generally observable in all fevers; but head-ache was not one of them! They were "*shivering, quick pulse, and heat,* varying in degree, at different times of the fever." By these three symptoms, Dr. Cullen also, who is no very despicable authority, as an observer of Nature, characterized the class pyrexia, as his celebrated definition of fever, which has never yet been improved upon, evinces—" post horrorem, pulsus frequens, calor major, *plures* functiones læssæ, viribus, presertim artuum imminutis." Where is *head-ache* here? In short, any man who ever felt in his own person, or accurately observed in others, the *ratio symptomatum* of fever, will confess that head-ache is very far from entering into the primary links of the morbid chain, much less of standing as the very foremost. A host of symptoms, indeed, indicative of *generally* impaired function in the nervous and vascular systems, precede the developement of actual *pain* in the head. There is a languor, a lassitude, and a general uneasiness of the whole body, with a corresponding inaptitude for exercising the functions of the mind. There is a sensation of cold, generally first along the spine, and radiating thence over the whole surface. The face and all extreme parts are at first paler and less full than usual; and so far from the brain and nervous system being in a state of *irritation or excitement*, they are in a state just the reverse. The *sensibility* of the whole body is *diminished*, as Dr. Fordyce has justly remarked, and this he has known to be in such a degree, " that even hot substances have been applied in such manner as to coagulate; nay, perform the chemical analysis of the part, without any sensation of heat having arisen in the mind of the patient." *First Diss.* p. 49.—Will any man, not biassed by some phrenitic hypothesis, maintain that the above phenomena are indicative of *phrenitis?*

But granting, for the sake of argument, what is not the fact, that head-ache were the first symptom; is this any proof of inflammation of the brain? There is scarcely a derangement of function, in any of the great organs or structures of the body, that will not cause head-ache. Let the function of the stomach, for instance, be disturbed, and see what an intense pain it will occasion in the head. How then can we expect that the centre of all sensation shall remain unaffected, when we have not merely "*plures* functiones læsæ," but, in fact, *omnes* functiones læsæ?

Dr. Clutterbuck having thus commenced in error, continues his course in uninterrupted delusion, inverting perpetually the order of nature and facts, by taking or mistaking causes for effects, and effects for causes. The inconsistencies into which our author is led, occasionally, by these false premises, are remarkable enough. Thus, after detailing the premonitory phenomena of fever, or such as precede the stage of reaction or excitement, he observes,

" There is yet no *remarkable general* disorder of the system, nothing that, *in strictness*, entitles it to the appellation of *fever*. Yet such symptoms constitute indubitably the *incipient* state of the disease." P. 18.

Thus an *indubitable* state or stage of fever is *not* fever! By a parity of reasoning, and by similar " medical logic," we might assert that, when this stage has passed away, and a state directly the reverse, viz. of excitement or reaction, has succeeded, it is not fever still, because it is only a more advanced stage of the disease! But, from what field of observation, during twenty years' study of fever, did Dr. Clutterbuck draw his data, when he asserts, that in the incipient stage of fever, there is "no remarkable *general* disorder of the system?" We fearlessly affirm, that the disorder of the system is then equally *general* as in almost any subsequent stage. What! when the whole muscular power of the strongest man is at once reduced to languor and tottering debility—when the functions of the mind are all deprived of energy—when digestion is almost completely suspended—when the secretions are locked up—in short, when the balance of the circulation, and the distribution of sensorial energy are evidently and universally unpoised, will any man tell us that—there is "no remarkable *general* disorder of the system?"

We are well aware, indeed, of the drift of this misrepresentation.* Dr. Clutterbuck would wish to persuade us that this (hitherto improperly considered) *general disorder* of the system, has but one "local habitation and name," inflammation of the brain; which inflammation is not only the cause of this stage, but of the subsequent one—which is *strictly entitled* to the appellation of fever. We shall therefore proceed to prove, 1st. That the phenomena attending the incipient stage of fever are *not* those which indicate inflammation of the encephalon; and 2dly. That the symptoms of this last disease do not correspond with those of fever in its incipient state.

A man, after exposure to the exhalations from certain marshy soils, shall exhibit all and each of the symptoms enumerated by Dr. Clutterbuck, as constituting indubitably the incipient state of that disease, which he endeavours to identify with inflammation of the brain; with this exception, that they will all be in a more intense degree than in common fever from contagion. These symptoms too, as in typhus, will be succeeded by a diametrically opposite class, constituting the stage of excitement; but, on the second day, they will all vanish, and the patient will be well, and able to transact every kind of his usual business. On the third day, they will return again; but in the course of three or four paroxysms, the disease will be completely cured—by what? Bark or Arsenic!

Will any man, even Dr. Clutterbuck himself, deny that

---

* We mean by this term, not a *wilful mis-statement*, but an erroneous exposition of facts.

this patient exhibited, over and over, the phenomena attending the incipient stage of typhus; aye, and most of those attending the second or excitive stage, and yet assert that he had every third day an inflammation of the brain, which inflammation was cured by bark or arsenic, without a single bleeding, or a purgative medicine?

But, on the other hand, does phrenitis exhibit those features which we see at the commencement of fever? We believe not. Phrenitis being an inflammation of an organ on which all sensation depends, will, of course, produce a more general fever than any other topical affection; but this fever corresponds with the *stage of excitement* in typhus or common fever; it has no similitude to that long period which precedes the reaction in idiopathic fevers. But what proves the distinction, beyond a doubt, is this; reduce the inflammation of the brain in phrenitis, and you *that instant* put a period to the fever, which is truly symptomatic; whereas, in fever from contagion, you may bleed, and reduce any local inflammation, yet the fever preserves its course, however mitigated in violence.

"Delirium vero febrile præviæ febri appenditur, solosque febricitantes adoritur; cur minime mirum si, hoc sedato, perstet febris, solitamque periodum absolvat. Aliter se res habet sub phrenitide, si enim resipiscant ægri illico sanati restituuntur, si excipias virium debilitatem."

Such were the sentiments of Lieutaud, and every man who has seen cases of phrenitis, either idiopathic, as in Coup de Soleil, or translated, as in gout, rheumatism, or erysipelas, will acknowledge the justness of Lieutaud's remark. If we look to the *post mortem* researches relative to the two diseases, we find the distinction verified by the most accurate and unbiassed observers. Dr. C. himself acknowledges, that in many fatal cases of fever, *inflammation* cannot be detected in the brain; but no man ever saw a patient die under phrenitis, without finding inflammation of the brain or its membranes, as unequivocally marked, as in pneumonia or carditis. Now, if the two diseases be of the same nature, and, as the Doctor says, have the same essential symptoms, why not the same appearances after death. Dr. Clutterbuck not only avoids all *minute* distinction between the *stages* of fever, as not at all suiting the homogeneity of his inflammatory hypothesis, but he actually blends, and most erroneously confounds them together, thereby exhibiting false portraits of the disease. Thus, speaking of that first stage of depression, which he says is not *strictly entitled* to the appellation of fever, he observes,

"There is a general listnessness, or indisposition to exertion, both mental and bodily. The external senses are *all*, more or less *disturbed*, being *generally*, at this period, *too acute*, and, at the same time depraved; so that light, and sound, and taste, and odour, that were before agreeable, become the contrary." P. 17.

Now every man of observation knows, that this *disturbance*, as Dr. Clutterbuck artfully designates it, consists, in the early stage, anterior to reaction, of *diminution*, not increase of sensibility in the organs of sense. The eyes are dull and inanimate; the hearing *thick*, as the patient expresses it; the taste and smell obliterated, as it were, for nothing is more common at these periods than the patient to assert that he can taste nothing, can smell nothing, &c. to the truth of which we adduced the testimony of Fordyce above. Thus, Dr. Clutterbuck has, knowingly or unknowingly, blended the two opposite states of depression and reaction under one general class of *disturbance*, and to *this* he has attributed *all* the phenomena of excitement, without noticing a single one on the opposite side. This passage, in short, exhibits one of the most glaring specimens of want of candour and impartiality, which we ever witnessed. A man of genius would try to explain away those phenomena which ran counter to his hypothesis, but Dr. Clutterbuck *suppresses* them, or clothes them in false colours.

Our author having thus confounded, or artfully eluded, all distinctions between the stages of primary oppression and subsequent reaction in fever, classing them both under the head of inflammation of the brain, continues to maintain the same erroneous consistency in his Therapeutics.

"I would observe, that the remedies which I have found to be unequivocally useful, in the treatment of the present epidemic are very few in number; and, in all respects the same as those employed in the ordinary cases of inflammation. They are blood-letting, vomiting, purging, and the digitalis, and occasional blistering."

Now we appeal to universal experience and observation, against the propriety of employing these remedies, especially blood-letting, in that stage which Dr. Clutterbuck has described as constituting the incipient state of fever, and before reaction has come on. It would, in nine cases out of ten, destroy, or greatly endanger the lives of the patients. Besides, we may in truth assert, that three in four of the common contagious and epidemic fevers of this country, would run their course, and terminate safely, *without any medical treatment at all.* Will Dr. Clutterbuck assert the same of phrenitis, pneumonia, gastritis, or enteritis? Here is another proof of the essential difference between idiopathic fever, and that where there is local inflammation as its cause. The late and present epidemic fever in Ireland has proved to a demonstration that the rate of mortality has been but little affected by the different, and sometimes diametrically opposite modes of treatment that have been employed. Did not a vast majority of practitioners, a few years ago, treat typhus fever with bark, wine, and opium, through all stages of the disease, and yet they did not lose *very many* more than the best practitioners of the present day. But what would have been the result, if they had treated phrenitis in this manner? Why, not one in twenty would have recovered.

We therefore disagree in toto with Dr. Clutterbuck, relative to the treatment in the incipient stage of fever, *anterior* to reaction, as mentioned in page 96. We might just as well bleed in the cold fit of an ague, as during the chilly, feeble state that precedes excitement in typhus fever. In respect to the method of treatment in the "second or active stage" (p. 98), we are as strenuous advocates for depletion as Dr. Clutterbuck, though upon very different principles, as will be presently seen.

The *post mortem* appearances in fever, not being at all in harmony with the phrenitic theory, we do not see them any where even alluded to throughout the volume! This, indeed, is in perfect consistency with the partial, garbled, and distorted representations of the phenomena of the disease, which are every where conspicuous in Dr. Clutterbuck's work; a line of conduct which must utterly, and for ever destroy our confidence in the arguments of a writer, be his talents what they may. We beg our readers to refer to the late papers on fever, in the Dublin Hospital Reports, and Transactions of the Association, as reviewed in our last number, particularly from page 546 to page 554, where the completest refutation of Dr. Clutterbuck's theory will be found, in the statements of Dr. Macartney, and Mr. Kirby, Professors of Anatomy in Dublin. These Gentlemen, in the midst of a capital where fevers were received, at the rate of *two thousand monthly*, into the hospitals, prosecuted their *post mortem* researches, under the guidance of science, candour, and fidelity—not under the blinding influence of preconceived hypothesis. And what was the result? Why, that

"The brain so constantly supposed to be the seat of inflammation, *rarely* exhibited the characters indicative of such a state; in some instances, this organ was much *paler* than usual; in a very few, amongst a great number of dissections, was there any evidence of sanguineous or serous effusion."—No. 4 of this Journal, p. 547.

We believe that no man, excepting Dr. Clutterbuck, would persist in maintaining so untenable a doctrine, in the face of all evidence and facts flowing from the symptoms, treatment, or pathology of fever.*

That the brain, as well as, and indeed rather than other organs, should suffer congestion or inflammation, *during* the great vascular derangements of the system, in idiopathic fever, is not to be wondered at. And as Dr. Clutterbuck appears to take a most confused and imperfect view of the subject, we beg permission here to throw out some hints that may help to elucidate the nature of congestion and inflammation; for even on these points, we think, Dr. Armstrong himself is somewhat obscure.

In health, and in ordinary states of the constitution, there is a *relative* proportion of blood in the arteries and in the veins; which, in fevers particularly, seems to be disturbed. Suppose, in the living body, the action of the heart was to suddenly stop; what would be the consequence? We know that the arterial system would free itself almost entirely of blood, by the organic and elastic contractility of the arterial coats. The whole mass of blood would thus be pushed into the veins and capillaries, particularly into the large interior trunks. This has been proved by experiments on animals, and by what we always see on opening bodies, however soon after death. Such an occurrence, in degree, takes place when we faint. The arteries go on lessening their diameters, simultaneously throughout the whole system, (not by contracting and dilating alternately, as has been erroneously supposed) till

---

* The following specimen of Dr. Clutterbuck's Cerebralogy, is worth recording,—" The extreme weakness of the lower limbs is principally felt when the patient attempts to stand; for when lying, he is scarcely sensible of his inability to walk. This is explained *by the gravitation of the effused fluid down the channel of the spine.*"

What fills up the space, in these cases, which the effused fluid occupied within the cranium, while the patient was supine? Was there a vacant space along the spinal marrow ready for the reception of the cerebral effusion, when the patient should get up to walk? We think these questions would require some ingenuity to answer!

But the explanation of the phenomenon is easily found in the greater facility with which the heart carries on the circulation in the horizontal posture, and the number of muscles which are necessarily thrown into action when the patient stands.

they almost entirely empty themselves. After a time, they also lose their organic power, and they relax again; lying, as we find them, with their sides in flat contact, and empty of blood. This cannot happen while the heart maintains its proper and healthy action, because it keeps the arterial system sufficiently replenished on one side, by throwing blood constantly into it; while it prevents undue turgescence of the venous system on the other, by perpetually abstracting blood from that side. Hence it appears that the HEART is the great engine which preserves the balance and harmony between the arterial and venous systems in the body. We have seen the consequence of its total cessation. What is the consequence of a preternaturally *diminished* action of the heart? Certainly, a *degree* of those phenomena abovementioned. In proportion to the weakened action of the heart, the proper proportion of blood in the arteries is less than in the veins. Here then, we see what takes place, when any of the causes of fever, for instance, contagion, is applied to the system. Among other phenomena, the action of the heart is diminished, and the volume of blood is proportionally accumulated in the veins. During this period, we have the symptoms which indicate the incipient stage of fever; or if miasmata were the cause, we have the cold fit of an intermittent. But when the reaction takes place—when the heart not only acquires its pristine power, but a far greater action than in health, then the scene entirely changes. The blood is rapidly transferred from the venous to the arterial system, the calibres of the arteries are increased, and their coats put completely on the stretch. Then it is, that the arterial capillaries swell, so as to redden the face, distend the surface, and raise the temperature. If the previous distention of the veins, in the brain, caused a heaviness, weight, or dull pain in the head, with watery eyes, the arterial distention now causes acute *throbbing* pain; red eyes; intolerantia lucis et soni, and a morbidly increased irritability of all the external senses; a state the very reverse of what occurred during the venous accumulation. Here we have a picture of the stage of reaction, or excitement, as it has been called.

Now, suppose there is some organ in the body weaker than the rest; its vessels may remain gorged with *venous* blood, even after the stage of excitement has commenced; and thus congestion may obtain: or, in the stage of reaction, the *arterial* system of the part may, from previous disposition or weakness, become *more than proportionally* distended; and, unless the action of the heart or the volume of the blood be reduced, *inflammation* may ensue.

The symptoms during life, and the appearances after death, all bear testimony to the truth of this doctrine, and prove that of ten people affected with typhus fever, there will not probably be more than three or four who, under common judicious treatment, shall exhibit unequivocal signs of any local inflammation at all; and of these three or four, there will not be two who shall have the same organs inflamed. If all the viscera were previously sound in structure, and free in function, the stage of excitement may run to a very great height, and no local inflammation ensue, because there is an equal and consentaneous resistance to the action of the heart throughout the system. It is very true, that the *functions* of all the organs will be deranged, and those of the brain and spinal marrow must be conspicuously so, from their universal influence over, and connection with, every part of the body. But derangement of *function* is one thing, and inflammation, which affects the structure, is another: and it is by confounding these together, that Dr. Clutterbuck has involved himself and many others in a labyrinth of perplexity and error. But we shall now return to points of a more practical nature.

Dr. Clutterbuck avers, and truly, that no "*known* state of the atmosphere, in regard to heat or cold, moisture or dryness, or *any other physical or chemical quality*," can be admitted to have given rise to epidemic diseases. And because the atmospheric quality is *unknown*, Dr. C. with his usual medical logic, infers, that it does not exist; and why? because the present epidemic has continued through a succession of seasons, and amidst a variety of atmospheric changes, "with little other difference than what we observe to be impressed upon diseases of all kinds by the influence of such causes." In the next page, Dr. C. asserts, that the idea of an epidemic being caused by *terrestrial* influence, is "destitute of all foundation." Thus having taken the power from *earth and air*, he must leave it, of course, to the "anger of the Gods," as the Greeks did at the Trojan war. The truth is, we know little of causes but by their effects. The matter of contagion itself is incognizable by the senses, yet we doubt not its existence. Hippocrates, Sydenham, and most of the ablest physicians of the present day, attribute the spread of epidemic diseases to aerial, or aerial and terrestrial influence combined; and many facts justify the conclusion. A few years ago, an epidemic fever travelled from Cape Comorin to the banks of the Cavery, a distance of nearly a thousand miles, and that precisely in the direction of the South-west Monsoon, affecting district after district, in regular succession, and sweeping to the grave 106,000 people! Can it be conceived that a fever would take such a course by mere accident, and independently of aerio-terrestrial influence?

Our author admits, and we perfectly agree with him, that from whatever general or unknown cause the fever may originate, it may "be afterwards propagated by *intercourse.*" He doubts the fact of scarcity of food producing an epidemic fever. It can only, we think, combine with other causes in propagating the disease, particularly through the medium of the *depressing passions,* which too generally accompany an empty stomach, especially in the person of John Bull.

Dr. Clutterbuck's observations on the *laws* of contagion are, in general, just, and without any pretensions to novelty. The same may be said of his remarks on the *pretion* of the fever itself. Early separation, and the destruction of the contagious virus, are the means, he thinks, most within our power. He leans to the doctrine of Dr. Carmichael Smith, respecting acid fumigations, but apparently with great doubt and hesitation. He advocates the policy, though he dreads the expence, of opening numerous places of reception for patients ill of fever.

*Treatment.* Here Dr. Clutterbuck is evidently puzzled, and not a little so, to make his doctrines quadrate with the different modes of practice which have been pursued in fever. For although his experience goes to confirm the general utility of the *depletory* system, yet he advances so many cautions and restrictions, relative to venesection in particular, that the hypothesis of cerebral inflammation being "the essence of fever," dwindles into nought!

> "In short, this it is which I am anxious to inculcate—not so much that we should *bleed* in fever (for that will require great discrimination); but that we should always bear in mind that it is *inflammation* we are treating, of which the *brain* is the seat, and that all our measures should be directed upon this principle." P. 73.

Now, we would ask Dr. Clutterbuck, if this seat of inflammation were any where else—in the lungs, liver, or intestines, would this great discrimination in bleeding, almost amounting to an abandonment of the measure, be necessary? Oh! but says Dr. Clutterbuck, the *brain* is a very particular organ, and *inflammation* there cannot be treated as inflammation elsewhere. But here again, we would ask the Doctor, if, in unequivocal phrenitis, the use of the lancet is not a *sine qua non*?

Dr. Clutterbuck divides fever into four stages:—

"The *first*, or incipient stage, as before described, is that in which the disease is not yet completely formed. The topical affection of the brain at this period is *sufficiently denoted by dull pains in the head*, restlessness, disordered sensations, and enfeebled muscular and mental powers; but there is little or no *pyrexia*, or febrile state of the system." P. 92.

Without stopping to show that inflammation of the brain is very *insufficiently* denoted by the above-mentioned symptoms, since at the moment that we are writing these lines, we experience them all, from a severe cold, we take this opportunity of entering our caveat against Dr. Clutterbuck's therapeutics in such cases. In this incipient stage of fever from contagion, or indeed from any other febrific cause, before reaction takes place, we do assert, that *blood-letting* is unwarrantable, notwithstanding the following sentiments of our author.

"Blood-letting to a moderate extent, when employed in this *incipient* stage of fever, will generally, as far as my observation goes, bring the disease to an almost immediate termination."

We hope we are not misunderstood on this point. The *incipient* stage of fever is *generally* applied to the early period of *reaction*, in which period, blood-letting is undoubtedly useful; but, during the stage of depression, from the operation of the febrific poison, and before the constitution begins to react, we are quite certain that the measure above mentioned would be fraught with pernicious consequences: and this opinion is not founded on hypothesis, but actual experience.

The following passage shows what degree of importance is to be attached to the preceding statement.

"The *necessity*, however, of employing so Herculean a remedy, at such a period of the disease, and before any alarming symptom has appeared, will, perhaps, be questioned. *Without doubt, it may often be safely dispensed with.*" P. 96.

Now, if this incipient stage of fever depended solely on *inflammation of the brain*, surely blood-letting could not safely be dispensed with often! Do we thus act or direct in inflammation of other internal organs? No. But the *fever* inflammation of the brain is of a peculiar kind, not yet perfectly understood! In other words, and in truth, it differs entirely from other inflammations, in symptoms, in treatment, and in *post mortem* appearances, and thus Dr. Clutterbuck has been studying twenty years, and writing two volumes about a *term*; to which, after all, he has not been able to affix a signification, or give any satisfactory explanation! He has not, in fact, gone one inch beyond his original motto.

> "To give to airy *nothing*
> A local habitation and a *name*."

In the second stage, or that of reaction, we go hand in hand with our author. Many of his directions are highly judicious—most of his observations are exceedingly interesting.

"If blood be largely taken at first, as from 20 to 30 ounces, and that within twenty-four hours, or little more of the attack, a single bleeding will often suffice for the cure. If only ten or twelve ounces be drawn, it is often found necessary to repeat the operation several times; till, in some instances, more than eighty or a hundred ounces have been taken away. By this mode, more blood is lost upon the whole, and the disease is more protracted. Numerous instances have occurred during the present epidemic, where a patient has been just able to crawl to the Dispensary, with fever strongly depicted in his countenance; tottering, and tremulous in all his movements; complaining of severe head-ache and pains in his back and limbs, with a tongue thickly coated; the pulse at the same time extremely feeble; and who, after a large bleeding, with other evacuations, has returned, on the following visiting day, free from complaint, and only moderately weakened." P. 102.

In cases of a worse description, and where the disease had run on three or four days unattended to, it generally required three or more moderate bleedings on successive days, to accomplish the cure. Dr. Clutterbuck thinks that this measure may be safely had recourse to any time within three or four days from the accession of the stage of excitement.

"When used with tolerable freedom within the period here mentioned, and *before* bad symptoms had made their appearance, *I have never once observed symptoms of malignity*, as they are called, to come on afterwards; neither the muttering delirium, the blackness of the mouth, the subsultus tendinum, nor the extreme prostration of strength, so often seen in cases that have been either left to themselves, or treated upon a different plan."

In many cases, Dr. Clutterbuck bled, with manifest advantage, at the end of the first week, but its effect is then, of course, to mitigate, not to cure the disease.

"Contrary to what is generally supposed, I think that the more the sensorial functions are disturbed—particularly the greater the delirium—the less confidence can be placed in this evacuation ......... The full and bounding pulse appears to me to be much more equivocal, as a reason for bleeding, than a small and contracted state of it. But where the pulse is extremely soft, and compressible with the slightest force, I hold blood letting to be altogether inadmissible." P. 109.

We were astonished to find a man of Dr. Clutterbuck's experience, object to *local bleeding* by leeches, because this measure acts in no other way than as abstracting a certain quantum of blood from the general circulation! He thinks that the same quantity of blood taken from a vein, will have even a better effect. Whether Dr. Clutterbuck has here, as in many other instances, viewed things through an inverted or distorted telescope, or has determined to oppose every idea that comes from certain other quarters of the medical horizon, we leave to the discrimination of a public that is not readily deceived upon such occasions. All we can say is, that his therapeutics are here wrong. The effects of local bleeding are not at all to be calculated on, or compared with the effects resulting from a detraction of a similar quantity from the general system. This is so well known to every tyro in the profession, that we shall not insult the understanding of our readers, by entering into a serious refutation of Dr. Clutterbuck's position.

*Emetics.* Dr. Clutterbuck conceives that it is not enough merely to discharge the contents of the stomach by emetics;—" the object is to produce *counter-irritation,* as the means of checking, and ultimately superseding the diseased actions that are going on in the system in general, but particularly in the brain." This is certainly a curious *rationale* of the operation of emetics. We were old-fashioned enough to suppose that their good effects resulted principally from their tendency to equalize the balance of the circulation, determine to the surface—promote perspiration, and open a number of the torpid secretions. But it appears that we were mistaken—it is by *counter-irritation* that they relieve. At this rate, the lytta, lunar caustic, or sulphate of copper, applied to the stomach, might prove more permanent *counter-irritants,* than antimony or ipecacuan.

" *Purging*, at the commencement of fever, is a remedy of the greatest utility, and which alone will often cut short the disease; like vomiting, it should be excited by active means, and repeated at short intervals, for three successive days. If the object is not then attained, the practice should be discontinued, for the excessive and long continued employment of drastic purgatives, may induce ulceration of the mucous membrane of the intestines, and thus add to the danger of the disease." P. 117.

Nevertheless Dr. C. recommends the bowels to be kept soluble throughout the disease, by mild means. The combination of emetics and cathartics, seems to our author, more powerful in cutting short fever, than either singly. He has not, however, found any medicine so efficacious as *elaterium*, in doses of an eighth or a quarter of a grain, two or three times in the twenty-four hours, and that for several days. The effect is increased by previous blood-letting.

In the stage of excitement, Dr. Clutterbuck considers digitalis as a medicine of great utility.

"I have often observed it, to all appearance, keep the fever effectually in check, so as to allow it to go quietly and mildly through its course, but without seeming to shorten its duration." P. 119.

Of the *cold affusion*, which our author supposes to act by *counter-irritation*, Dr. C. has no experience. Of blisters he has little opinion, and of opium less; of antimonials he speaks very slightingly in his *book*, though we heard him speak highly of them in a large assembly.

Dr. Clutterbuck informs us, that when inflammation has continued for a considerable time, and when the general strength is materially reduced, either by the treatment had recourse to, or by *the continuance of the disease*, a moderately *cordial and soothing* plan of cure is often the best. What are those inflammations? We believe there is but *one man in England*, besides Dr. Clutterbuck, who will give *stimulants*, while any inflammation continues in an internal organ, especially the brain—and that man is Dr. Charles M'Lean.

The foregoing piece of bad therapeutics, however, paves the way for an attempted explanation of the principle on which opium, wine, camphor, ammonia, &c. are exhibited in *fever*. But we are not to be caught with chaff. The plain fact is, that Dr. Clutterbuck can give no rational explanation why inflammation of the brain requires wine, opium, and other stimulants.

Dr. C. is "very indifferent as to a patient's taking food in diseases, *however active or inflammatory*; provided the food is of a simple kind, and taken with real appetite." The benefit, indeed, of good eating in fevers, is elucidated by a most ingenious hypothesis, equal to any branch of the great phrenitic doctrine.

"By putting the *organs of supply* into an *active* state, by the moderate use of food, the *attention of the system* (to speak figuratively) is divided, and the disease proceeds with less *activity.*" P. 128.

Now, while we are ready to grant that it is very natural for this system *personified* to direct her attention to the quarter where *good cheer* is going forward, rather than to scenes of inflammation and disease, we have no hesitation in condemning both principle and practice. In fevers and active inflammations, however, these "*real appetites*" are among the airy nothings which our author has pressed into his service; and which, we verily believe, exist only in a distempered brain. There is a craving for food sometimes observed in yellow fever, just before the fatal termination; but, with this exception, the loss of appetite is just as regular and constant a symptom of fever, as head-ache, quick pulse, hot skin, or any other phenomenon by which the existence of fever is recognized.

Of the third stage of fever, or collapse, Dr. Clutterbuck's experience does not enable him to say any thing satisfactory. He enters, however, into a long train of cavilling against Dr. Armstrong's doctrine of venous congestion.

"It would be difficult (says Dr. C.) to find elsewhere so many *unfounded notions*, within the compass of so few words; *and it would be not less so, to find an example of their obtaining equal currency in so short a space of time.*"

Aye! there's the rub. The doctrine of *venous congestion* has obtained more credit in ten months, than the *phrenitic* theory in ten long years. During the latter period, not a single writer of note has stepped forward to second Dr. Clutterbuck's motion, that fever is inflammation of the brain; while scarce a month passes wherein the most authentic documents do not appear, destructive of the Doctor's hypothesis.

Of the attack which is here made on Dr. Armstrong's work we shall say nothing,

"The blood of Douglas can defend itself."

We have already far overstepped our limits; but we hold it a duty, of paramount obligation, to examine, with strictness, a *doctrine* which bears so directly on practice, as that of Dr. Clutterbuck. We have examined his arguments with great freedom, and without any very fastidious delicacy, because we are quite satisfied, that all unbiassed men will agree with us in the charge of *partiality*, which we have preferred against the work—a fault which is extremely inimical to the progress of medical science in particular. No talent, rank, or reputation of an author, shall ever deter us from speaking our sentiments on such occasions.

Cum tabulis animum censoris sumet honesti. Hor.

---

We now come to Dr. Percival's volume, which is an enlarged and improved republication of his REPORT in the Transactions of the Irish Colleges, of which we gave an analysis in our first number. On this account, we shall not enter so deeply into the contents of the work as its merits deserve, lest we should repeat what had been before touched upon.

Dr. Percival, on more mature deliberation, is disposed to class typhus in the following manner; viz. Typhus gravior, mitior, and mitissimus. Each of these species, he observes, commences with some congestive or inflammatory diathesis; each may decline with stupor, sub-delirium, and death; and the appearances on dissection complete the analogy. He considers that, whether fevers owe their origin to contagion or any other febrific cause, they become capable of propagating themselves afterwards from individual to individual.

"They differ from fevers arising from simple *local inflammation*, in many important particulars; but in none more remarkably than the sudden failure of mental and voluntary power—the tendency to perform a certain cycle of morbid changes in definite periods, and the power of propagating their kind in healthy subjects by contagion." P. 63.

In a subsequent passage, at page 91 of the Essay, we have these words.

"With respect to the brain, [in typhus mitior] I have examined many cases in which no deviation occurred from the due or ordinary condition of that viscus."

These testimonies are not very favourable to Dr. Clutterbuck's theory. From Dr. Percival's chapter on the Pathology of Fever, we shall select the following passage.

"From whatever cause, or combination of causes, typhus fever originates in the body, the sensorial and circulating systems are primarily disturbed. The failure of mental and bodily power, with obtuse or depraved senses, are so invariably attended with disturbed balance of the sanguiferous and secerning systems, that an intimate connection must subsist somewhere between these morbid phenomena. All the incipient symptoms of typhus denote enfeebled energy of the heart and arteries, with its usual attendant, venous congestion. Whether this venous congestion, in the sinuses of the brain, and throughout the cerebral structure, be the cause, or remotely the effect of sensorial oppression;—whether also it occasion the pain so commonly felt in the occiput and the spine, the sickness of the stomach, and the torpor of the bowels, may not be strictly demonstrable. The facts, however, are prominent, and more or less strongly associated. The pale, leaden, or venous hue, which attends the incipient stage of fever, the oppression of the præcordia, the sighing or yawning to afford relief to the pulmonary circulation, the laboured or obscure movements of the heart, the torpor of the arterial capillaries, whether of the skin or mucous surface of internal cavities, demonstrate the disturbed balance of the arterial and venal systems. In some parts of the body, as the spleen, the liver, the brain, where a peculiar provision is made for venous plethora, this condition seems to obtain, not merely at the onset, but more or less throughout the course of typhus fever. No parts indeed appear to be exempt from it, at the commencement of the fever; and where the diurnal or advanced stages of arterial excitement are feebly developed, venous plethora, local or general, prevails to the last. Most commonly, however, the incipient congestion of the veins is followed by inordinate actions of the heart, and the vessels depending upon it. Hence an inflammatory diathesis supervenes, marked by phenomena very different from those just described; viz. a full and florid surface, shining eyes, pains in the forehead, throbbing pulse, hurried respiration, and increased heat and sensibility. After a while, these symptoms decline, as their precursors, whether the diurnal or the entire cycle of the fever be regarded. Yet as I have just observed of the venous plethora, so also of the arterial excitement, a perfect or equal subsidence does not take place in all the parts or organs of the body. Some viscus or tissue retains the inflammatory condition excited by the fever, when other parts are freely disengaged. The mucous membrane of the bronchi very commonly falls into this condition; which, as in peripneumonia notha, is accompanied with venous congestion throughout the substance of the lungs. The like coincidence of venal or arterial repletion is often observed in the villous coat of the primæ viæ, the several textures of the brain, and the serous tissues in many parts of the body." 100.

This is enlarged and enlightened pathology, forming a striking contrast with the narrow, contracted cerebralogy of Dr. Clutterbuck and his followers. The outline of Dr. Percival's *methodus medendi* will be found in our first number, pages 66, 7, 8, 9, 10, to which we beg to refer. This little volume is extremely creditable to the talents of Dr. Percival, and we strongly recommend its perusal to all those who have not possession of his original Report, on which the present volume is founded.

Notwithstanding our closing limits, we must still say a few words on the *prevention* of fever.

Dr. Dickson, of Clifton, in a very logical little tract on this subject, has justly observed, that "the diffusion of an evil often tends to lessen the individual sense of its magnitude;— the number implicated has the delusive effect of wonderfully reconciling the community to even *evitable* misfortunes; and of furnishing each with a specious pretext for leaving the remedies to others." The danger, however, is now becoming so general, that people, to preserve *themselves*, begin to think of guarding others from a disease, which, Phœnix like, rises more powerful from its own ashes—*viresque acquiret eundo!* It is therefore culpable and unmanly to gaze in apathy at the enemy while advancing to our gates, without attempting to meet him on the plain. It is very true, that over the original sources, and many of the exciting causes of epidemic fever, we have but feeble control; yet we can disarm it of much of its power, by checking its dangerous tendency towards successive reproduction. The way to do this is obvious to all ranks of the profession; namely, separation, ventilation, and cleanliness. It is needless to expect any good effects from legislative interference; every town where fever prevails, must legislate and act for itself. The rich man should come forward with his purse; the clergy with their influence; the profession with their personal services, in this general work of humanity. Temporary hospitals should every where be erected, no matter how simple in their internal economy. To these all cases of fever should be immediately transferred, when pronounced as such by the proper medical officer; while a benevolent band should every where enrol themselves for the purpose of cleansing and purifying the apartments and clothes of the sick thus removed to hospital. These things should even be done in *anticipation*. It is in peace that we prepare for war; and so it is in health that we should construct our antidotes, and preventive checks of disease. The early and prompt removal of half a dozen fever patients to a house of recovery, on the first appearance of its visitation in a town, would, in all human probability, *prevent* eventually *one hundred cases* of the disease.*

* For further particulars on this head we refer to the excellent little tracts of Dr. Dickson, Dr. Porter, and Mr. James Parkinson.

P. S. Before finally terminating this protracted, but important article, we shall take a slight glance at a work which has excited much attention on the continent. It is on Typhus Fever, and from the pen of Professor Hildenbrand, of Vienna. A very copious analysis of this book has just been published in a respected cotemporary Journal.*

Professor H. considers contagious typhus as a febrile disease, *sui generis,* like small pox, which, "*from a determinate cutaneous eruption,*" belongs to the class exanthemata. The constant symptom, in every stage, is *stupor,* with delirium or typhomania—"accompanied with a more or less evident affection of the liver." He thinks it is not, in its own nature, either inflammatory, nervous, or putrid; but assumes all these characters occasionally. It may arise from the reception of its own specific contagion, or be *generated* in the course of some other febrile affection, which newly generated disease is contagious from thenceforward. The German Professor divides typhus into eight stages. The 1st that of *Infection,* is probably but a few seconds duration; during which, the morbid poison penetrates into the system of the individual, unaccompanied, he thinks, by any particular sensation. The second, or "*stage of opportunity,*" is that in which those premonitory symptoms of indisposition are observed, which precede any febrile affection. This stage, he believes, never lasts longer than seven, nor less than three days. In this stage, he thinks, the disease may sometimes be arrested by cold affusions, &c. but not afterwards. The 3d is the *stage of invasion,* in which the actual febrile paroxysm begins.

"A creeping sensation over the head and back is first felt, and then the decided shivering takes place, accompanied with the other concomitants, paleness of the surface, cutis anserina, &c."†

This stage rarely exceeds twelve hours.

The 4th is the *inflammatory stage,* corresponding with our reaction. In this stage the typhous eruption appears.

"This consists of a red, spotted efflorescence, having a great resemblance to the mottled appearance which the skin of a healthy person presents, when exposed to a slight degree of cold. Among the spots are interspersed small papulæ, not so prominent as those of the miliary eruption, but rather like those of measles. With

---

* Quarterly Journal of Foreign Medicine and Surgery, No. 3.
† Journal of Foreign Medicine and Surgery, No. 3. p. 225.

this, miliariæ and petechiæ (dependent on accidental causes) are frequently conjoined." *ib.* 226.

This stage closes with the 7th; and the 5th or *nervous stage,* begins on the eighth day of the fever. The heat of the surface is now remarkably increased, and communicates a burning sensation to the hand. The cutaneous eruptions, with the exception of the petechiæ, disappear. The epidermis becomes dry and shrivelled, the patient's torpor and indifference are increased. In this stage the sensorium suffers in a peculiar manner. We have tremors, subsultus tendinum, slight convulsions, &c. with deafness, impaired vision, smell, and taste. The patient dreams without being asleep, and talks deliriously. He has generally one predominant false impression or train of ideas. This stage lasts during the 8th, 9th, and 10th days; at the end of which, a stronger than usual evening exacerbation takes place, and lasts for a few hours, when a gentle perspiration or evacuation from the bowels is succeeded by an evident remission. This remission is most considerable on the eleventh day; but on the 12th and 13th, it is again supplanted by increased febrile heat, and aggravated affection of the nervous system.

The *sixth stage* is that of crisis. At the end of the 13th day, a most severe exacerbation takes place; the heat is more glowing; the arteries pulsate more strongly; the brain is more affected, and a state of sopor occurs. On the 14th, the skin relaxes, " the external apertures of the body seem to open and to be freed from spasm, and the decisive moment arrives." Some evacuation from the external or internal surfaces marks the crisis.

The *seventh stage* is that of abatement of all the symptoms; and the eighth is that of convalescence.

Professor Hildenbrand conceives that the new contagious miasma is not generated until the appearance of the eruption, and that it is in its most active state during the permanent dryness of the skin in the nervous stage of the disease. He thinks that fomites cannot retain the poison longer than three months in an active state.

" The opinions regarding the proximate cause of typhus are innumerable. *That of Marcus, which makes it inflammation of the brain, is erroneous, and merely substitutes a part for the whole;* for the cause might, with equal justice, be said to be an inflammation of the intestines, &c." *Journal of Foreign Medicine, p.* 234.

Upon the whole, the Professor thinks, that the *secreting membranes,* though not actually inflamed, very nearly approach to it, in typhus; and to this succeeds the affection of the nervous system. In the inflammatory stage, local inflammations, sub-inflammations, or congestions, may grow out of the general excitement of the system.

The following extract will give a tolerable view of the German Professor's *methodus medendi*.

"We first find that, in simple ordinary typhus, the vital powers, of themselves, are sufficient to effect the most perfect recovery, and that this happens only after a certain period of time, and after precise changes in the system; and then that this disease has a course so determinate, that no method has hitherto been discovered by which it can be successfully shortened, much less cured—that death alone can shorten it; or, that interrupted typhus produces death.

"Things being thus constituted, nothing remains for the practitioner but to adopt the indirect method of cure, to await the operations of Nature, by which this cure is effected—to support her in these; to set aside all impediments, in order that the vital powers may exert their beneficial influence, in a free and undisturbed manner, until the disease be surmounted, and the contagious process completely terminated." P. 289.

In the third or stage of invasion, nothing should be done but give tepid diluents, and preserve a moderate warmth in bed. In the fourth, or inflammatory stage, our business is to guard any weakened organ from the effects of high general excitement. Emetics, he recommends; and, if the lungs are severely affected, to precede its use by venesection. But even in this stage, he trusts principally to mild diaphoretic or diluent drinks, with, occasionally, the neutral salts, to stimulate the mucous membranes and clear away morbid secretions.

"Whoever adopts, in the ordinary course of typhus, a very active practice, such as blood letting, purgatives, and stimulants, will disturb the progress and crisis of the disease."

Speaking of blood-letting in particular, the German Professor says,

"In many, and certainly in most cases, it is pernicious, not merely in the nervous, but in the inflammatory stage. In mild, regular typhus, occurring even in plethoric habits, it is a superfluous remedy; but a highly aggravated inflammatory character, or dangerous local inflammation indispensibly requires it. If it be omitted in such cases, the local affection ends unfavourably." 240.

He condemns severe purging, as "it has the effect of drawing the fluids from the skin, and occasioning colliquative diarrhœa."

In the ulterior stages, blisters, camphor, arnica, and small doses of wine, are highly spoken of.

Having thus presented our readers with a view of Professor Hildenbrand's doctrines and practice in typhus, it only remains to make a very few reflections of our own.

It is quite evident that the fever described as typhus by our author, does not assimilate with what we call typhus here. Where is the exanthematous eruption in our typhus? It appears that Professor H. has greatly erred in making typhus obey such regular laws as the exanthematous fevers observe. We see nothing to justify such doctrine in this country. Nevertheless, there can be no doubt but the Professor's descriptions are drawn correctly from life; and this leads to the inevitable conclusion, that typhus, as well as other diseases, is so modified by climate and locality, that the therapeutics of one country are inapplicable to the diseases of another. Although, therefore, Professor Hildenbrand's work may prove a classic production for the meridian of Vienna, it will not do here at all—an observation, indeed, which applies to most, even of the best continental writings—particularly as regards the *treatment* of disease. To those concerned in the *foreign* trade, therefore, we would strongly advise the more frequent importation of *pathology,* in preference to continental *therapeutics,* which do not suit the English market.

